# Supplementary material for: Pigmentation and TYRP1 expression are mediated by zinc through the early secretory pathway-resident ZNT proteins
Source: Commun Biol. 2023 Apr 18;6:403. doi: 10.1038/s42003-023-04640-5 (PMC10113262; doi:10.1038/s42003-023-04640-5)
Supplement: Supplementary file 2 — Supplementary Information [file 42003_2023_4640_MOESM2_ESM.pdf]

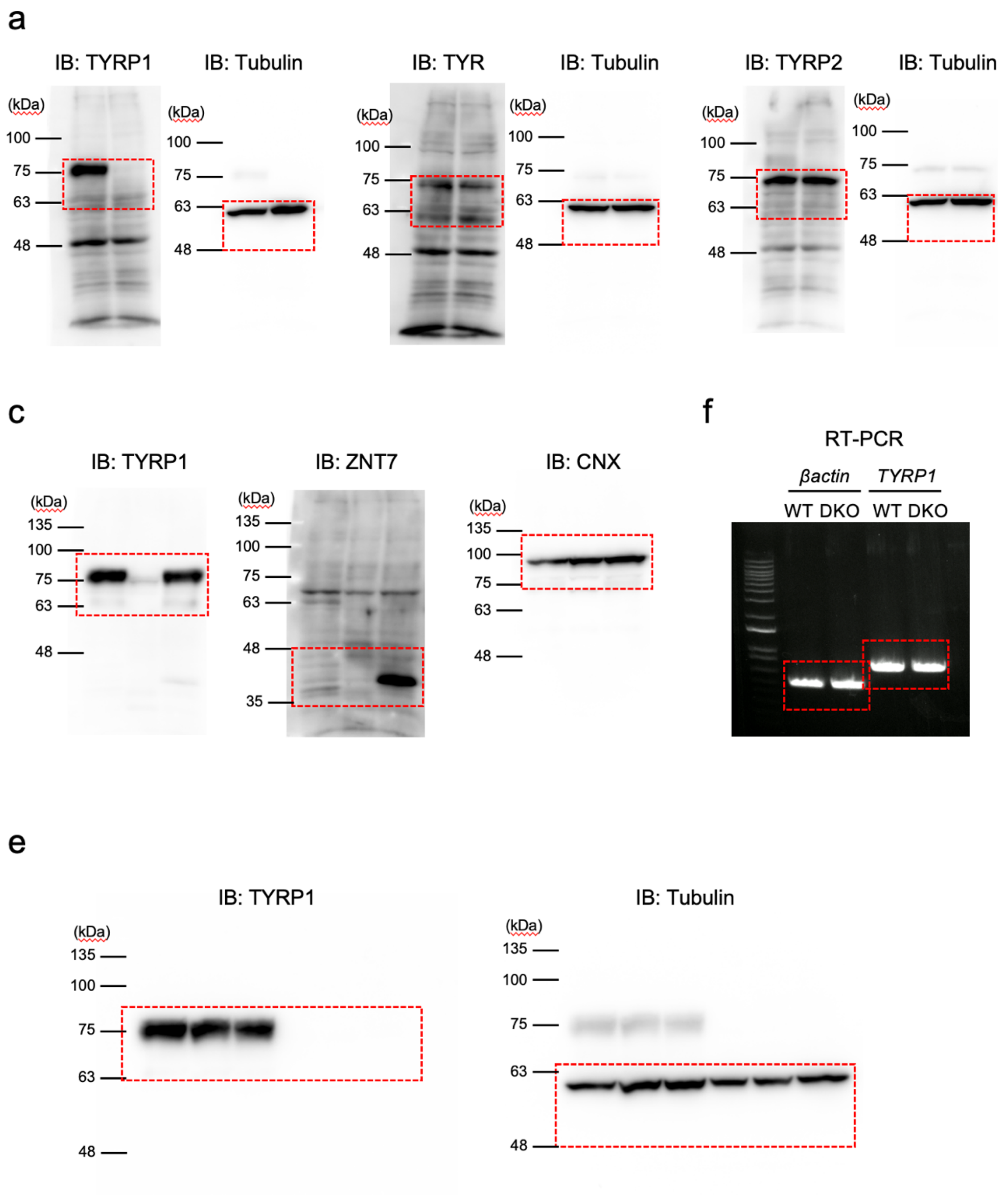

Supplementary Figure 1. Full-length immunoblot and RT-PCR images used in Figure 3. The panel used is boxed. In a, c, and e, the same blot was used sequentially (after stripping) for detection in each composite figure. The molecular weights of the marker proteins are indicated on the left of the immunoblot images.

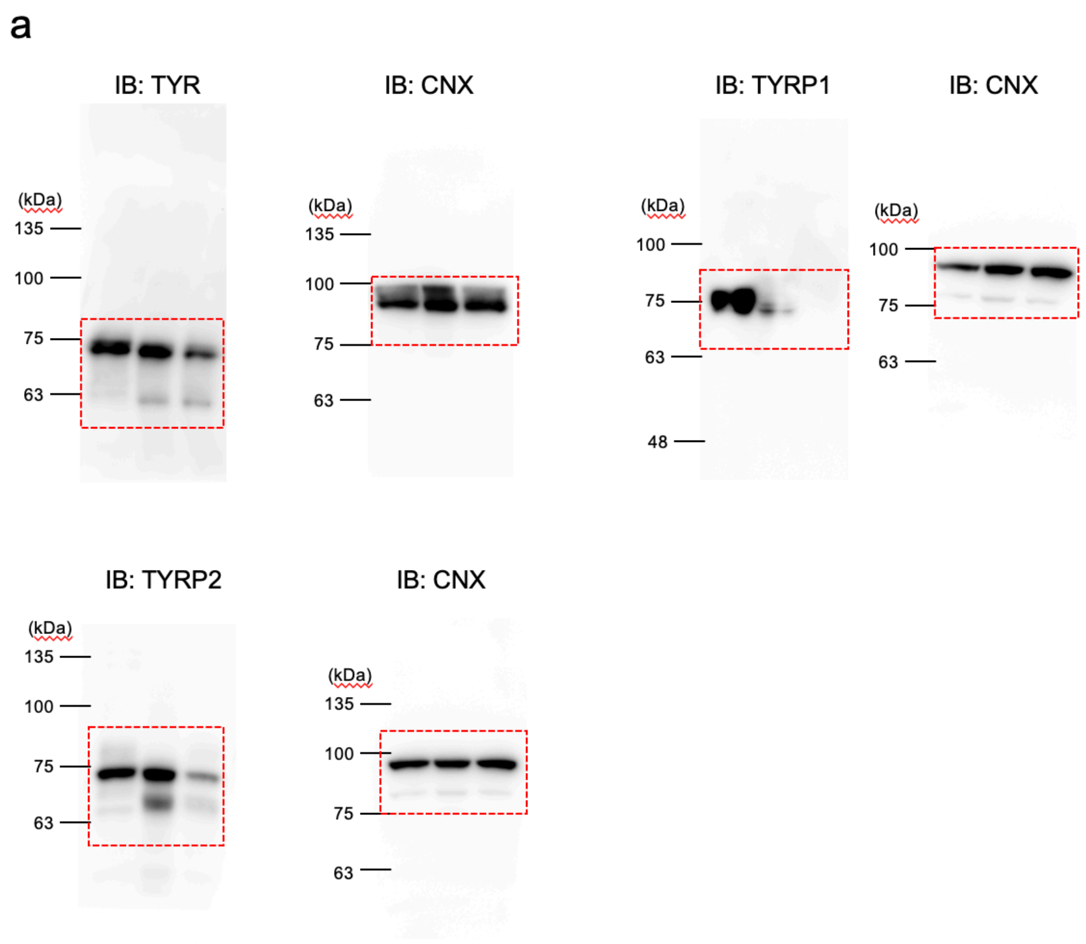

Supplementary Figure 2. Full-length immunoblot images used in Figure 4. The panel used is boxed. The same blot was used sequentially (after stripping) for detection in each composite figure. The molecular weights of the marker proteins are indicated on the left of the immunoblot images.

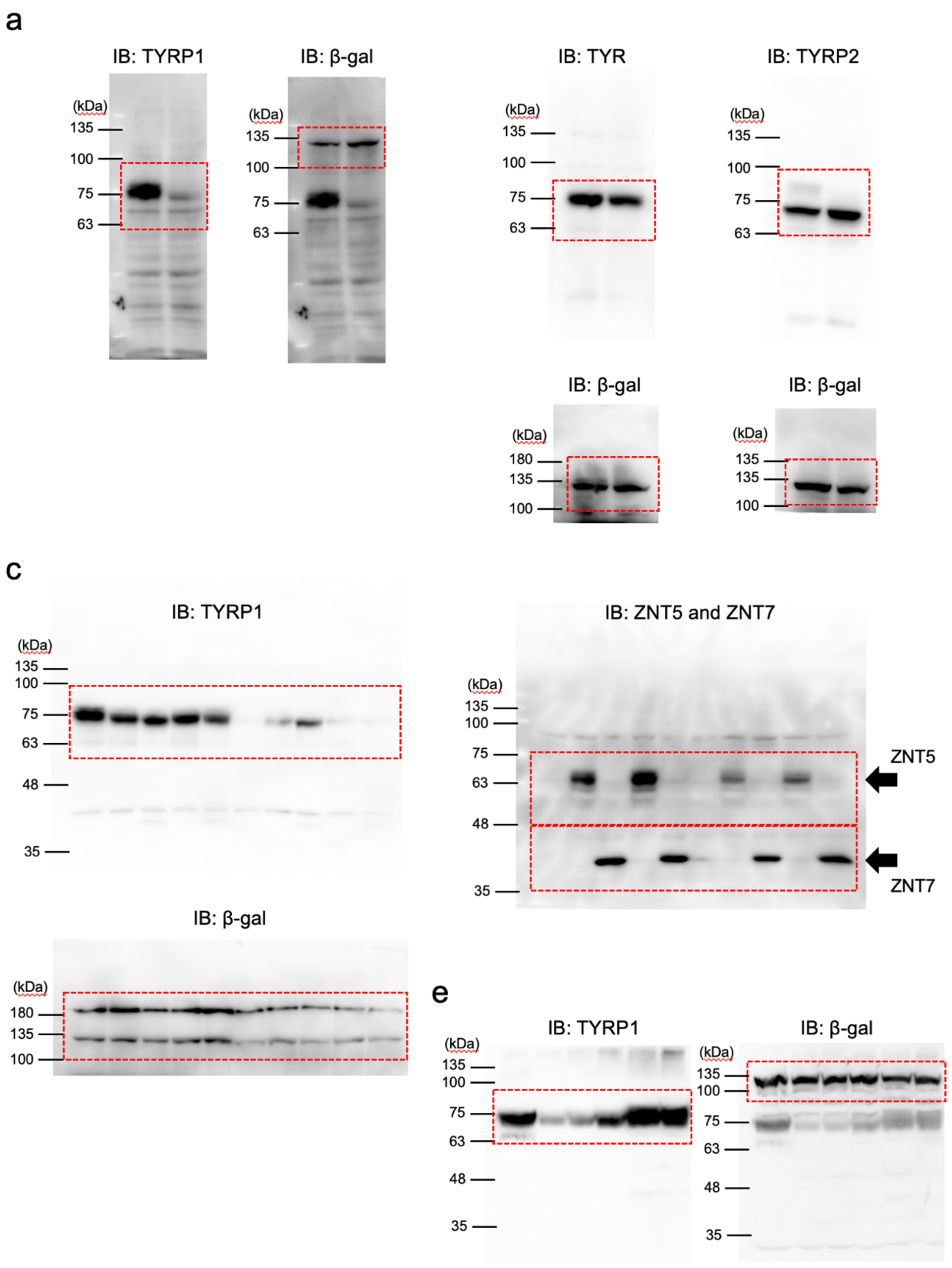

Supplementary Figure 3. Full-length immunoblot images used in Figure 5. The panel used is boxed. The same blot was used sequentially (after stripping) for detection in each composite figure. The molecular weights of the marker proteins are indicated on the left of the immunoblot images.

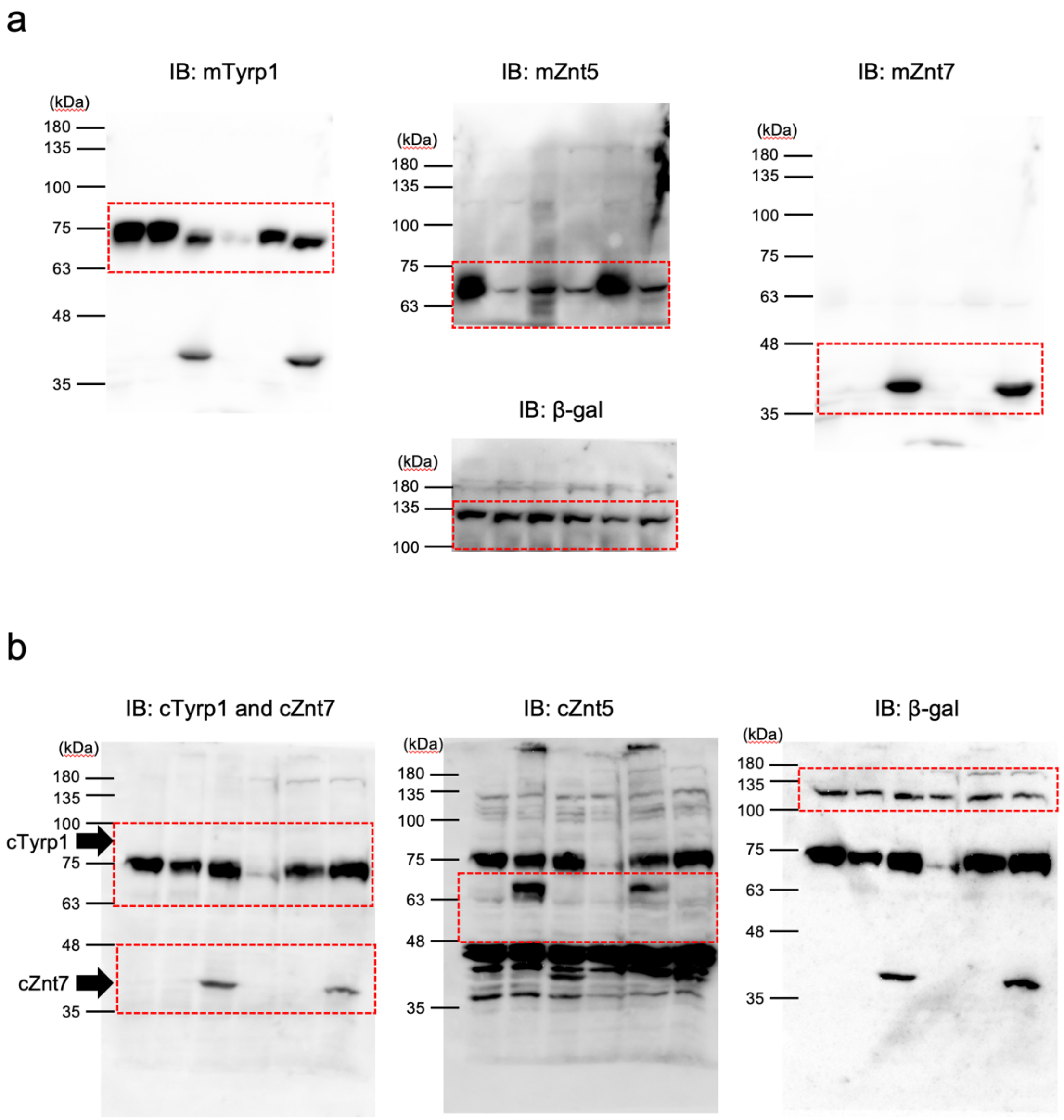

Supplementary Figure 4. Full-length immunoblot images used in Figure 6. The panel used is boxed. The same blot was used sequentially (after stripping) for detection in each composite figure. The molecular weights of the marker proteins are indicated on the right of the immunoblot images.

a

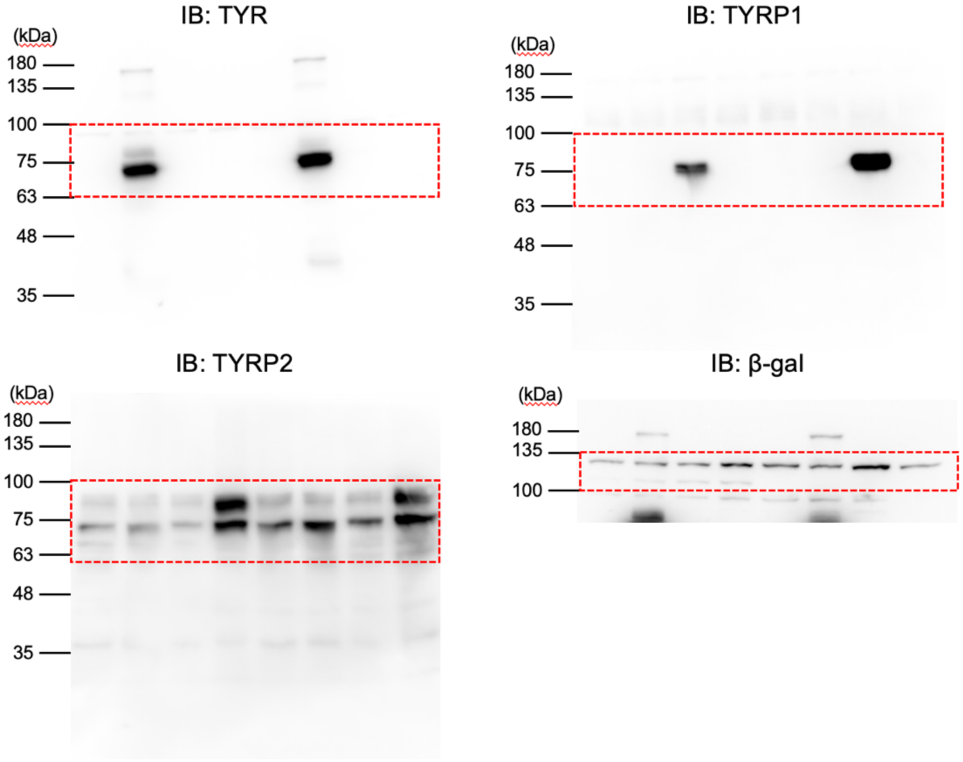
IB: TYR
(kDa)
180
135
100
75
63
48
35
IB: TYRP1
(kDa)
180
135
100
75
63
48
35
IB: TYRP2
(kDa)
180
135
100
75
63
48
35
IB: β-gal
(kDa)
180
135
100

c

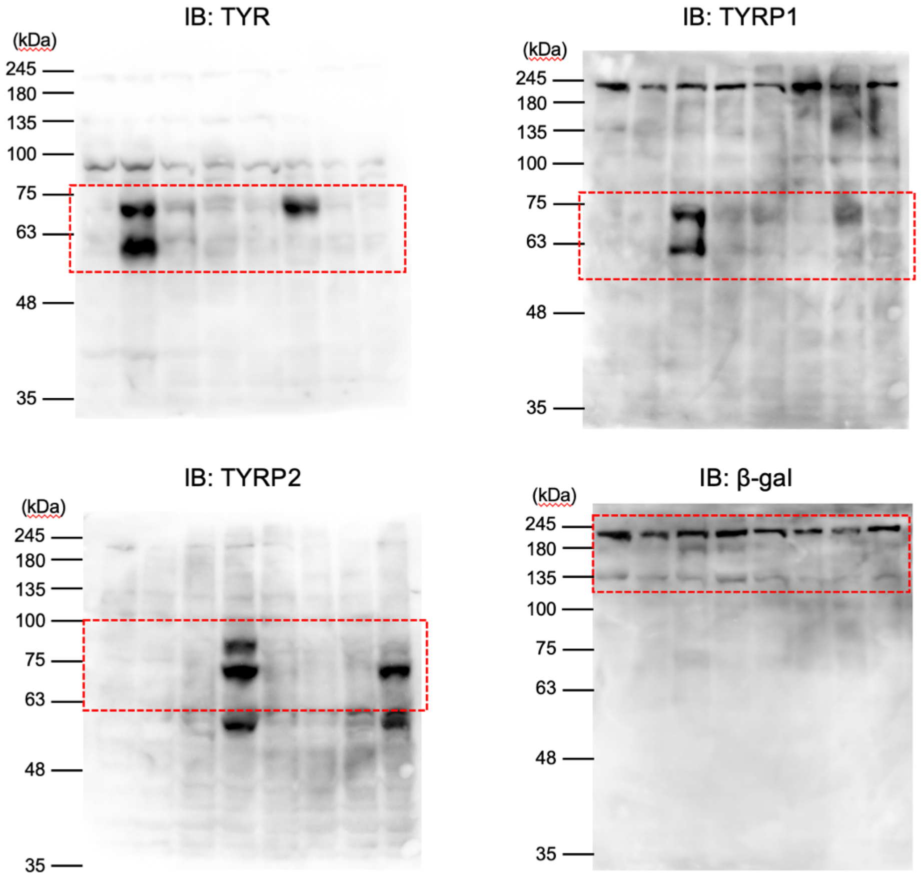
IB: TYR
(kDa)
245
180
135
100
75
63
48
35
IB: TYRP1
(kDa)
245
180
135
100
75
63
48
35
IB: TYRP2
(kDa)
245
180
135
100
75
63
48
35
IB: β-gal
(kDa)
245
180
135
100
75
63
48
35

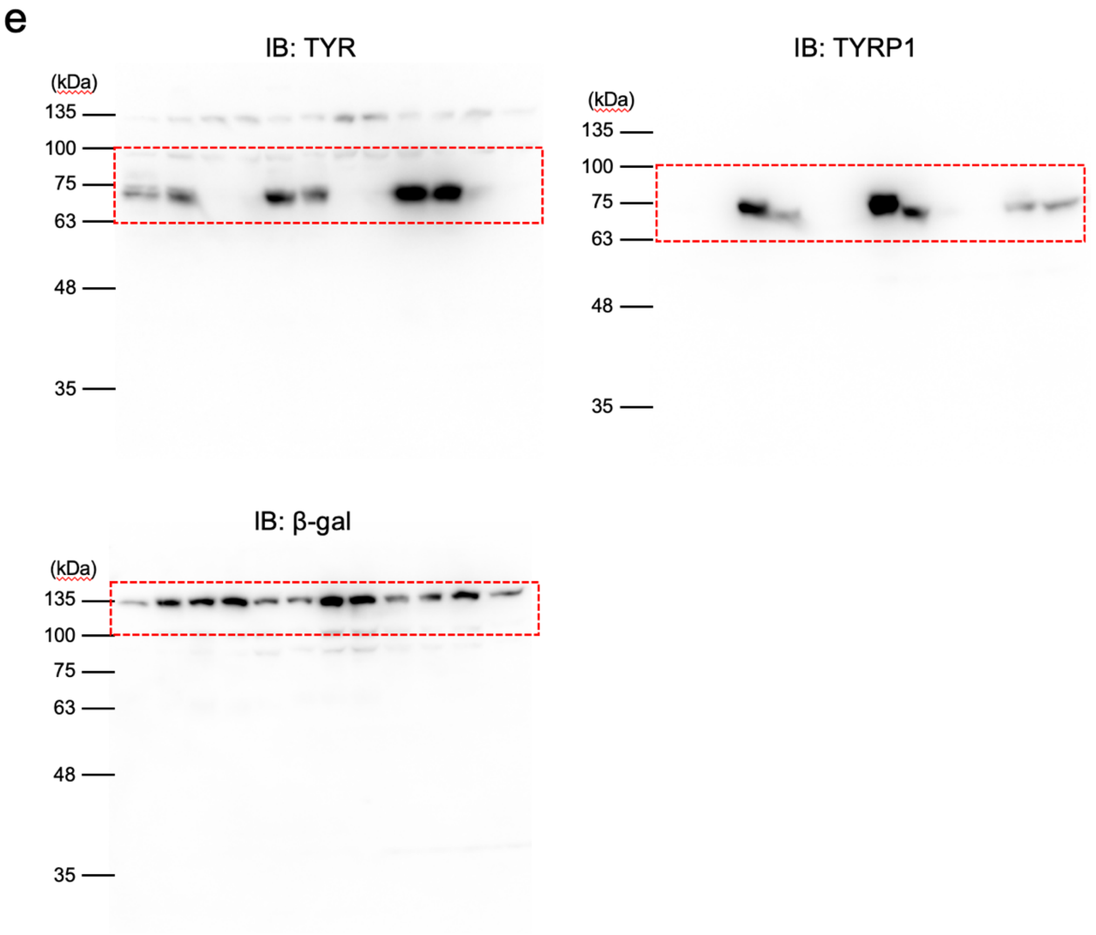

Supplementary Figure 5. Full-length immunoblot images used in Figure 7. The panel used is boxed. The same blot was used sequentially (after stripping) for detection in each composite figure. The molecular weights of the marker proteins are indicated on the right of the immunoblot images.

Supplementary Table 1. Primers used for genomic PCR to confirm gene editing in Mewo and SK-MEL-2 cells.

Gene	Forward primer (5′ to 3′)	Reverse primer (5′ to 3′)
ZNT5	TGTGTGAAATAGGGTTTCTAATGTAAGTGG	TAATCTTTGAGCTTAATTCCATTCAGTATT
ZNT7	GTGAAGAAAGGAGCATGTGAACTG	CAGAGGTAGGTAACAAAGGTAGGAC
TYRP1	CTTCAAGGCCATGTGGCCAATGTAA	AAGATTCTGAAAGGGTCTTCCCAGC
ATP7A	TCTGTATTCCTGTAATGGGGCT	AGCTGAAAATAAACCTTGCCTG

Supplementary Table 2. Primers used in RT-PCR analysis.

Gene	Forward primer (5′ to 3′)	Reverse primer (5′ to 3′)
TYRP1	GCTGCCTGTGACCAGAGGGTTCTCATAGTC	GGTCATACTTTCCCGTGGGGTCACTGTAAC
βACTIN	CATGTACGTTGCTATCCAGGCTGTGCTATC	GTCATACTCCTGCTTGCTGATCCACATCTG
