# Supplementary material for: Pigmentation and TYRP1 expression are mediated by zinc through the early secretory pathway-resident ZNT proteins
Source: Commun Biol. 2023 Apr 18;6:403. doi: 10.1038/s42003-023-04640-5 (PMC10113262; doi:10.1038/s42003-023-04640-5)
Supplement: Supplementary file 3 — Description of Additional Supplementary File [file 42003_2023_4640_MOESM3_ESM.docx]

**Description of Additional Supplementary File**

File name: Supplementary Data 1
Description: The source data behind the graphs in the paper
